# Cemented versus uncemented hemiarthroplasty in patients with displaced femoral neck fractures

**DOI:** 10.12669/pjms.321.8461

**Published:** 2016

**Authors:** Mohsen Khorami, Hamidreza Arti, Amir Aghaie Aghdam

**Affiliations:** 1 Mohsen Khorami, Assistant Professor, Department of Orthopedic Surgery and Trauma Research Center, School of Medicine, Ahvaz Jundishapur University of Medical Sciences, Ahvaz, Iran; 2 Hamidreza Arti, Professor, Department of Orthopedic Surgery and Trauma Research Center, School of Medicine, Ahvaz Jundishapur University of Medical Sciences, Ahvaz, Iran; 3 Amir Aghaie Aghdam, Resident, Department of Orthopedic Surgery and Trauma Research Center, School of Medicine, Ahvaz Jundishapur University of Medical Sciences, Ahvaz, Iran

**Keywords:** Hemiarthroplasty, Femoral Neck Fractures, Uncemented, Cemented, Displaced

## Abstract

**Objective:**

This study compared functional outcomes and preoperative between cemented and uncemented bipolar hemiarthroplasty in patients older than 65 years with subcapital displaced femoral neck fracture.

**Methods:**

Fifty one patients with displaced femoral neck fracture were enrolled in this study. Twenty nine patients underwent uncemented bipolar hemiarthroplasty and 22 underwent cemented bipolar hemiarthroplasty. Physical examination and radiographs were performed at the first and sixth months after operation and results were recorded. The patients’ pain and function were measured with Visual analogue Scale and with Harris Hip Score (HHS), respectively and then compared with each other.

**Results:**

The mean duration of follow up was 18.9 and 19.5 months in the cemented and uncemented groups, respectively. All patients were followed up for at least 6 months. Mean operation and bleeding times were longer in the cemented group compared to the uncemented group (P>0.05). The mean pain score was significantly less in the cemented group compared to the uncemented group (P=0.001). Hip functional outcome based on HHS was more in the cemented group (P= 0.001). The intraoperative and postoperative complication rate was higher in the uncemented group (P<0.05).

**Conclusion:**

Although higher rates of intraoperative bleeding and surgery time were seen with cemented bipolar hemiarthroplasty in older patients with femoral neck fracture compared to uncemented bipolar hemiarthroplasty, cemented bipolar hemiarthroplasty can cause less complications and improve patients’ function in less time.

## INTRODUCTION

Femoral neck fracture is more common in females and the mean age of onset is 81 years. That with disability and mortality imposes high health care costs on the health system. The risk of femoral neck fracture is about 40-50% in females and 13-22% in males.^1^

Epidemiologic studies have recognized several risk factors for femoral neck fracture, including BMI<18.5, Insufficient sunlight, low activity, smoking, history of osteoporosis related fracture, positive history of hip fracture in his or her mother and treatment with corticosteroid. The usual cause of this fracture is a simple fall in which force is transmitted from greater trochanter to femoral neck.^2^ Other mechanism is leg external rotation with increased force on the capsule and iliofemoral ligament.^3^ Intracapsular femoral neck fractures account for about 50% of hip fractures. The union rate is low because of low blood supply and intracapsular situation; it is also sometimes associated with femoral head necrosis and delayed segmental necrosis. In recent years, the improvement of health services and increased life expectancy has dramatically increased the incidence of this type of fracture.

It is estimated that the incidence of femoral neck fracture with a change of lifestyle will grow from 1.66 million in 1990 to 6.25 million in 2050 in the world^1^. The treatment of displaced femoral neck fracture in people over 60 years is hemiarthroplasty or total hip arthroplasty depending on the activity level before fracture. Hemiarthroplasty is recommended in people with routine activities and THA in highly active people.^4^

There are different types of cement and uncemented bipolar prosthesis. This prosthesis has an articular surface between the head and shell and articular surface between the acetabulum and shell. Tow joint prosthesis are likely to reduce wear and protrusion to the acetabulum. We can use orthopedic cement for stability of stem into femoral canal to increase the stability of stem and decrease loosening rates; in contrast, this can lead to complications such as increased intraoperative bleeding and embolism.^5^

## METHODS

In this prospective study, in a simple convenience sampling, all patients with displaced femoral neck fracture older than 65 years old who were referred to Imam Khomeini hospital in Ahvaz Jundishapur University of Medical Sciences from 20011-1-12 were enrolled. Seventy three patients with femoral neck fracture underwent bipolar hemiarthroplasty. Twenty two of patients (2 of them died and 20 did not take part in follow up period thus) were excluded. All patients were selected for performing a cemented or uncemented bipolar hemiarthroplasty based on Dorr classification.^6^ Exclusion criteria were pathological fracture, simultaneous intertrochanteric fracture, uncontrolled diabetes, severe cardiovascular disease, respiratory disease, uncontrolled neurologic disease and renal disease.

After approval of the ethical committee of Ahvaz Jundishapur University of Medical Sciences and obtaining written informed consent from all patients, 29 patients underwent uncemented and 22 of them underwent cemented bipolar hemiarthroplasty by an orthopedic surgeon. Zimmer femoral component was used for all patients. Follow up was performed in the first, and sixth month (4 and 12 weeks after surgery) after the operation. The intensity of pain (based on visual analog scale), hip function (according to Harris hip score), radiological signs of patients x-ray (the presence or absence of acetabular erosion loosening of prosthesis) and postoperative complications were recorded. All data including age, sex, type of treatment, intraoperative bleeding volume, the mortality rate (during surgery until discharge) and treatment costs were collected by a questionnaire and check list and analyzed by SPSS.^19^ Frequency, ratio, mean and standard deviation of variables were calculated, to compare quantitative variables for which chi-square was used. Binary variables were analyzed by Fisher’s exact test, and continuous outcomes were analyzed with the use of the Student’s t-test (two-tailed). Survival and the duration of hospitalization were further analyzed with use of the Kaplan-Meier method. P<0.05 was considered significant for all analyses.

## RESULTS

Over a period of two years, 73 patients with femoral neck fracture in Ahvaz Imam Khomeini Hospital of Ahvaz Jundishapur University of Medical Sciences underwent bipolar hemiarthroplasty. Twenty two of patient (2 of them died and 20 did not take part in follow up period) thus excluded. Fifty one patients who fulfilled the inclusion criteria and data from hospital records and follow up were evaluated. Of these, 19(37%) were men and 32(63%) were female. The mean duration of follow-up were 18.9 and 19.5 months in cemented and uncemented groups, respectively, and none of the patients were followed up for less than 6 months. The mean age was 79(70-92) years with cemented group and 71.7(65-76) years old in uncemented group. The mean of operation time was 95 minutes in cemented group and 75 minutes in uncemented group. The mean of intraoperative bleeding volume was 330cc and 258cc in cement and uncemented groups, respectively (P>0.05). Duration of admission was 11 days in cement group and 10 days in the uncemented group that there were no significant differences with each other (P>0.05) (Table-I).

**Table-I:** Demoraphic data of two group’s patient

Variable	Uncemented (N=29)	Cemented (N=22)	p-value
Age(year)	71.7 (65-76)	79(70-92)	0.45
Right side (No)	14 (48%)	10 (45.5%)	0.4
Left side (No)	15 (52%)	12 (54.5%)	0.23
Male (No)	17(59%)	2(10%)	0.04 *
Female (No)	12(41%)	20(90%)	0.02 *
Duration of hospitalization (day)	10(3-14)	11(5-17)	0.67
Operative time (minutes)	75	95	0.001 *
Intraoperative Blood Loss (ml)	285	330	0.9

* Significant at P = 0.05.

The meaning of pain, according to VAS criteria was 2.6 ± 0.8 after one month and 1.6 ± 0.6 after 6 months in cemented group that was 3.2 ± 1 and 2.6 ± 0.9 in un cemented group, respectively and there were significant differences (Table-II).

**Table-II:** Mean±SD degree of residual pain at the follow-up assessment.

Postoperative Week	Uncemented (N=29) visual analog scale (VAS)	Cemented (N=22) visual analog scale (VAS)	p-value
4th week	3.2±1	2.6±0.8	0.02 *
24th Week	2.6±0.9	1.6±0.6	0.001 *

* Significant at p = 0.05.

Hip functional outcome, according to HHS in cement group at one month were poor in six patients, moderate in five patients, good in eight patients and excellent in three patients. At six months the result was poor in four patients, moderate in three patients, good in eight patients and excellent in seven patients. The mean of HHS in cement group was 83 in 6 months (Table-III).

**Table-III:** Hip functional outcomes in cement group, according to HHS at one six months.

Postoperative Week	Excellent (90-100)	Good (80-89)	Fair (70-79)	Poor >70
4th week	3 (13.4%)	8 (36.4%)	5 (22.7%)	6 (27.3%)
24th Week	7 (31.8%)	8 (36.4%)	3 (13.6%)	4 (18.2%)

Good+Excellent(6 months):68.1% Mean HHS:83.

After surgery in uncemented group at one month functional outcome was poor in ten patients, moderate improvement was seen in nine patients, good in six patients and excellent functional outcome was observed in four. At six months the functional outcome was poor in five patients, moderate improvement in thirteen. (Table-IV) There was significant differences between the two groups. Oveall Hip functional outcome was 68.1% in cemented and 37.8% in uncemented group at 6 month (Table-V)

**Table-IV:** Hip functional outcomes in uncemented group, according to HHS at one and six months.

Postoperative Week	Excellent (90-100)	Good (80-89)	Fair (70-79)	Poor >70
4th week	4(13.7%)	6(20.6%)	9(31%)	10(34.5%)
24th Week	5(17.2%)	6(20.6%)	13(44.8%)	5(17.2%)

Good + Excellent (6 months):37.8% Mean HHS: 78.

**Table-V:** Hip functional outcome in cemented and uncemented group, according to HHS at 6 months

Group	Good + Excellent	Excellent	Good	Fair	Poor
Cemented	68.1%	7(31.8%)	8(36.3%)	3(13.6%)	4(18.2%)
Uncemented	37.8%	5(17.2%)	6(20.6%)	13(44.8%)	5(17.2%)

Intraoperative and postoperative total complication rate was 21.5% in cemented group and 31.5% in uncemented group which was higher significantly (Table-VI) (P<0.05).

**Table-VI:** Intraoperative and postoperative total complication rate in cemented and un-cemented group.

Complications	Noncemented		Cemented		P Value

	Number	Percent	Number	Percent	
Cardiovascular	1	3.5%	3	13%	P>0.05
Upper respiratory infection	1	3.5%	0	0%	P>0.05
Superficial and deep wound infection	1	3.5%	1	4.3%	P>0.05
Urinary tract infection	1	3.5%	1	4.3%	P>0.05
Postoperative fracture	0	0%	0	0%	P>0.05
Intraoperative fracture	4	14%	0	0%	P>0.05
Reoperation	0	0%	0	0%	P>0.05
Dislocation	1	3.5%	0	0%	P>0.05
Total	9	31.5%	5	21.5%	P>0.05

## DISCUSSION

Femoral neck fracture is more common in older people, and the mortality rate is high. about preferred treatment of femoral neck fracture is still being debated.^1^ Because of high complications and mortality rate with nonoperative treatment, recent studies are on the introduction of operative treatment that has the lowest cost and complications and results in better function in older people. Because of the need for reoperation other available methods of surgical treatment hemiarthroplasty is more preferred.^6^ This method is performed with unipolar and bipolar prosthesis. The bipolar prosthesis causes less erosion and protrusion in acetabulum because of movement between metal head and polyethylene cover and movement between metal cup and the acetabulum (outer bearing). Moreover, femoral neck length and head size are variable and can be converted to THA. Therefore some studies have shown better outcomes with this prosthesis for femoral neck fracture treatment in elderly. Recently, some studies have evaluated the indications for performing hemiarthroplasty with or without the use of cement which had different results.^7^,^8^

Therefore, in this prospective study, we compared cemented and uncemented hemiarthroplasty in patients who underwent hemiarthroplasty in the last two years in this center. We compared the Harris hip score (HHS) in both cemented and uncemented hip arthroplasty and showed significant improvement in patients benefited from a cemented method some studies in patients which were followed for six months. The mean HHS was 83.1. Functional results in cemented group were excellent in 33%, good in 43%, fair in 17% and poor in 7% which is similar to the results of our study. The mean duration of hospitalization was 15.3(4-29) days which in our study was 10(3-17) days. The patients in some studies were painless in 70% had minimal pain in 20% and moderate pain in 10% after 6 months.^7^,^8^

In two valuable review studies, cemented group’s patients had less pain at three months after surgery and better mobility after six months.^9^,^10^ The incidence of residual pain at 6 months after surgery were 23.6% and 34.4% in cemented, uncemented groups, respectively, which was statistically significant (Relative risk 0.69, 95% CI 0.53-0.90:0.007).^10^-^12^ However, in some studies, although complications, intraoperative and postoperative fractures and subsidence in considerably more common in uncemented group, but the mean of visual along scale was noted significantly different between the two groups.^6^,^9^,^13^ In our study the mean pain score was less in cemented group and it was statistically significant (p<0.05).

Several studies have showed that there is no significant difference between two groups as regards mortality, need for reoperation and postoperative complications^9^,^10^,^14^-^16^ although Carpintero et al. in a systematic review has showed that the meantime of surgery and bleeding volume was more in cemented group^17^ that is similar to our study.

In some studies that were performed to compare cemented and uncemented groups, showed that the need of reoperation, intraoperative complication and survival rate of implant is more in cemented method than uncemented group.^18^,^19^ Although in Gjertsen et al study the risk of revision hemiarthroplasty in cemented group was 2.1 times higher compared to uncemented. (95% confidence interval 1.7 to 2.6, p<0.001).^8^

In our study, the mean operation time was 95 minutes in cemented group and 75 minutes in uncemented group, respectively. The mean bleeding volume was 330cc in cemented group and 285cc in uncemented group (p>0.05).

Deep vein thrombosis, pulmonary emboli, fat emboli, displacement or fracture of femoral neck, superficial and deep infections and foot drop are hemiarthroplasty postoperative complications.^10^,^12^,^14^ In our study the total complication rate was 21.5% in cemented group and 31.5% in uncemented group, which was significantly higher in cemented group (p<0.05).

In Lo et al study, the intraoperative and postoperative complication rate were 63 cases in cemented group and 228 cases in uncemented group which was significantly higher in uncemented group (p<0.05)^18^ that is similar to our study.

## CONCLUSION

Despite high intraoperative bleeding and time of surgery in elderly patients with femoral neck fracture, the cemented bipolar hemiarthroplasty can cause less complications and increase patients function levels in less time compared to uncemented bipolar hemiarthroplasty.
